# How to Design, Create, and Evaluate an Instruction-Tuning Dataset for Large Language Model Training in Health Care: Tutorial From a Clinical Perspective

**DOI:** 10.2196/70481

**Published:** 2025-03-18

**Authors:** Wojciech Nazar, Grzegorz Nazar, Aleksandra Kamińska, Ludmila Danilowicz-Szymanowicz

**Affiliations:** 1 Department of Allergology Faculty of Medicine Gdańsk Medical University Gdansk Poland; 2 Faculty of Medicine Gdańsk Medical University Gdansk Poland; 3 Department of Cardiology and Electrotherapy Faculty of Medicine Gdańsk Medical University Gdansk Poland

**Keywords:** generative artificial intelligence, large language models, instruction-tuning datasets, tutorials, evaluation framework, health care

## Abstract

High-quality data are critical in health care, forming the cornerstone for accurate diagnoses, effective treatment plans, and reliable conclusions. Similarly, high-quality datasets underpin the development and performance of large language models (LLMs). Among these, instruction-tuning datasets (ITDs) used for instruction fine-tuning have been pivotal in enhancing LLM performance and generalization capabilities across diverse tasks. This tutorial provides a comprehensive guide to designing, creating, and evaluating ITDs for health care applications. Written from a clinical perspective, it aims to make the concepts accessible to a broad audience, especially medical practitioners. Key topics include identifying useful data sources, defining the characteristics of well-designed datasets, and crafting high-quality instruction-input-output examples. We explore practical approaches to dataset construction, examining the advantages and limitations of 3 primary methods: fully manual preparation by expert annotators, fully synthetic generation using artificial intelligence (AI), and an innovative hybrid approach in which experts draft the initial dataset and AI generates additional data. Moreover, we discuss strategies for metadata selection and human evaluation to ensure the quality and effectiveness of ITDs. By integrating these elements, this tutorial provides a structured framework for establishing ITDs. It bridges technical and clinical domains, supporting the continued interdisciplinary advancement of AI in medicine. Additionally, we address the limitations of current practices and propose future directions, emphasizing the need for a global, unified framework for ITDs. We also argue that artificial general intelligence (AGI), if realized, will not replace empirical research in medicine. AGI will depend on human-curated datasets to process and apply medical knowledge. At the same time, ITDs will likely remain the most effective method of supplying this knowledge to AGI, positioning them as a critical tool in AI-driven health care.

## Introduction

### Background

Why is high-quality data the cornerstone of modern artificial intelligence (AI)-driven health care? Reliable data enable AI algorithms to assist medical professionals in making evidence-based decisions, reducing the likelihood of errors and improving patient outcomes [1-5].

Accurate datasets are the foundation for developing large language models (LLMs) and deep learning models based on the transformer architecture [1,6-12]. This technique enables the model to learn the structure, grammar, and nuances of language, as well as factual knowledge and patterns of reasoning [9-12]. Examples of state-of-the-art all-purpose transformer-based models include generative pretrained transformers (GPTs) from OpenAI, Gemini and Gemma models from Google DeepMind, and LLaMA LLMs from Meta [8,13,14].

Foundation LLMs are trained on vast amounts of data encompassing millions of samples from diverse sources such as books, studies, and websites [8,13,14]. The goal is to improve the model’s generalization capabilities. This means enabling the model to apply learned patterns and knowledge to a wide range of unseen inputs, ensuring it produces accurate, meaningful, and contextually relevant outputs rather than merely memorizing the training data [8,13,14]. Among various types of datasets used to train LLMs, instruction-tuning datasets (ITDs) used for instruction fine-tuning (IFT) has emerged as a pivotal technique in enhancing LLM performance and generalization capabilities across diverse tasks [8,9,15]. For example, OpenAI researchers report that, even though the InstructGPT model with 1.3 billion parameters has more than 100 times fewer parameters than the original 175 billion-parameter GPT-3, its outputs were preferred over GPT-3 [8]. An ITD contains examples of task instructions paired with corresponding responses, enabling models to understand better and follow human-like directives [8,9,16]. This structured training approach not only improves task-specific accuracy but also enhances the LLM’s ability to generalize knowledge across multiple domains, sometimes including even domains that were not extensively covered in the original dataset [8,9,15,16].

Given the importance of ITDs, preparing high-quality ITDs in health care is critical. Such datasets may facilitate the formation of robust LLMs capable of addressing the nuanced requirements of complex medical questions, where precision, adaptability, and context-specific understanding are essential. In the clinical setting, instruction-tuned LLMs may support clinical decision-making and reduce the risk of medical errors. It will ultimately improve clinically relevant outcomes like increased patient safety and reduced hospitalizations, intensive care unit admissions, or deaths [4,5,17-20].

### Aim

This paper provides a guide on the key principles of designing, creating, and evaluating ITDs for training LLMs in health care applications.

### What Is an ITD?

ITDs are used during the IFT of LLMs [8,9,12,21]. These datasets typically consist of instruction-input-output (IIO) triples, such as an appropriate instruction combined with a question and its corresponding answer [8,9,21]. The primary aim of IFT is to improve a model’s ability to comprehend and execute various instructions, particularly those relevant to the medical domain, ultimately developing a specialized medical LLM [12,21,22].

ITDs differ significantly from the general datasets used for supervised fine-tuning (SFT). The key distinction between databases used for SFT and IFT lies in their objectives and methodologies. SFT primarily seeks to integrate domain-specific medical knowledge into a general LLM by continuing pretraining, enhancing the model’s understanding of medical texts [8,12]. It creates a “medical foundation model” [12,21]. Conversely, IFT focuses on improving the model’s responsiveness to instructions and tailoring its outputs to align with specific guidance and human-like responses rather than emphasizing token prediction accuracy as in ST [12,21]. Usually, the IFT follows the SFT [8,9,12,21].

While SFT relies heavily on the volume of training data, IFT prioritizes the quality and diversity of the data. In general, IFT improves the performance of the baseline SFT model [7,16,21,22]. Recent research has explored combining these approaches to build robust medical LLMs, leveraging the strengths of both techniques for better overall accuracy [7,16,21,22].

## Considerations for Designing ITDs in Health Care

### Data Sources

**Figure 1:**
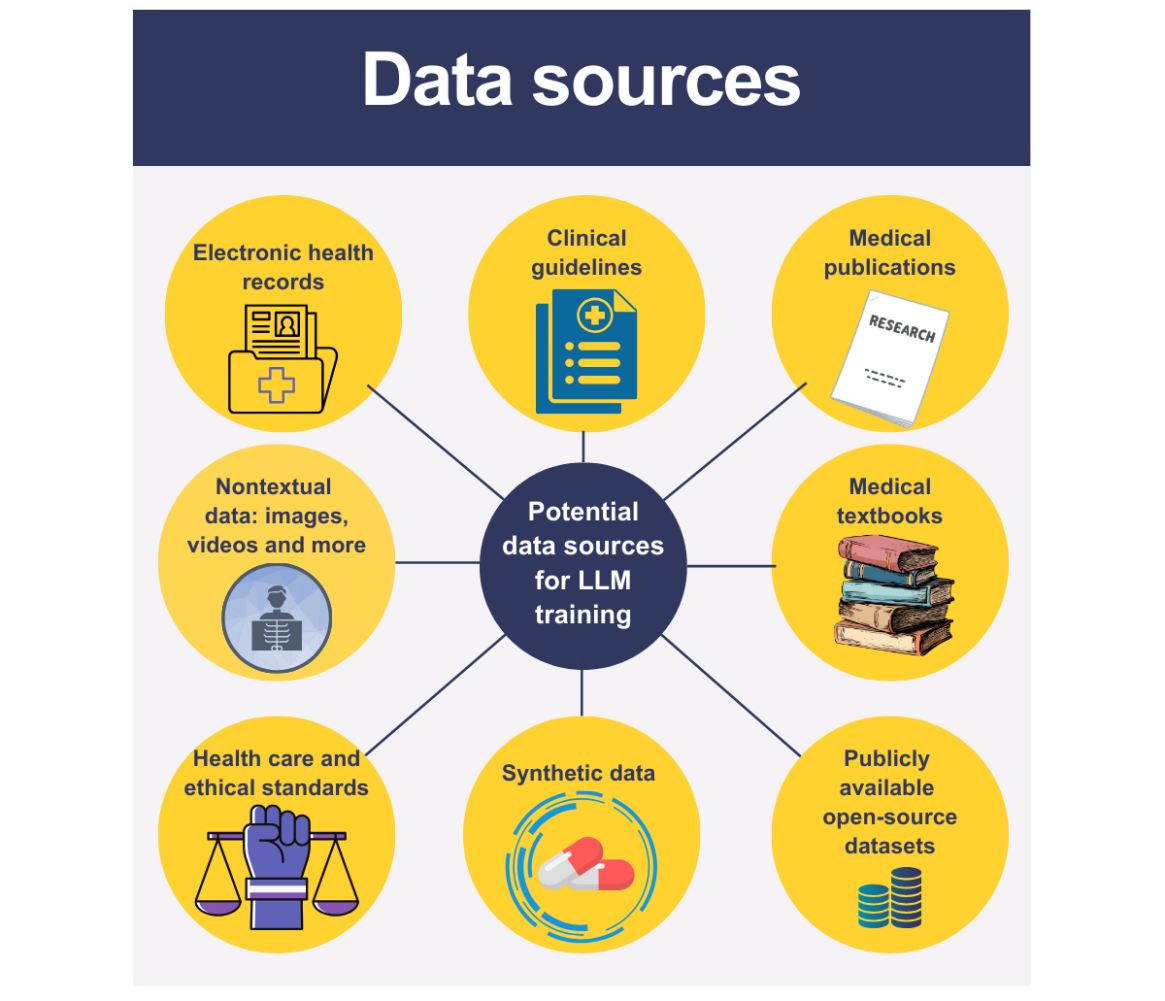
Potential data sources for instruction-tuning datasets. LLM: large language model.

Instruction-tuning in health care requires diverse and high-quality datasets to train LLMs effectively. Potential sources for such data are presented in Figure 1 and described in detail in Multimedia Appendix 1.

#### Key Features of a Well-Designed ITD

**Figure 2:**
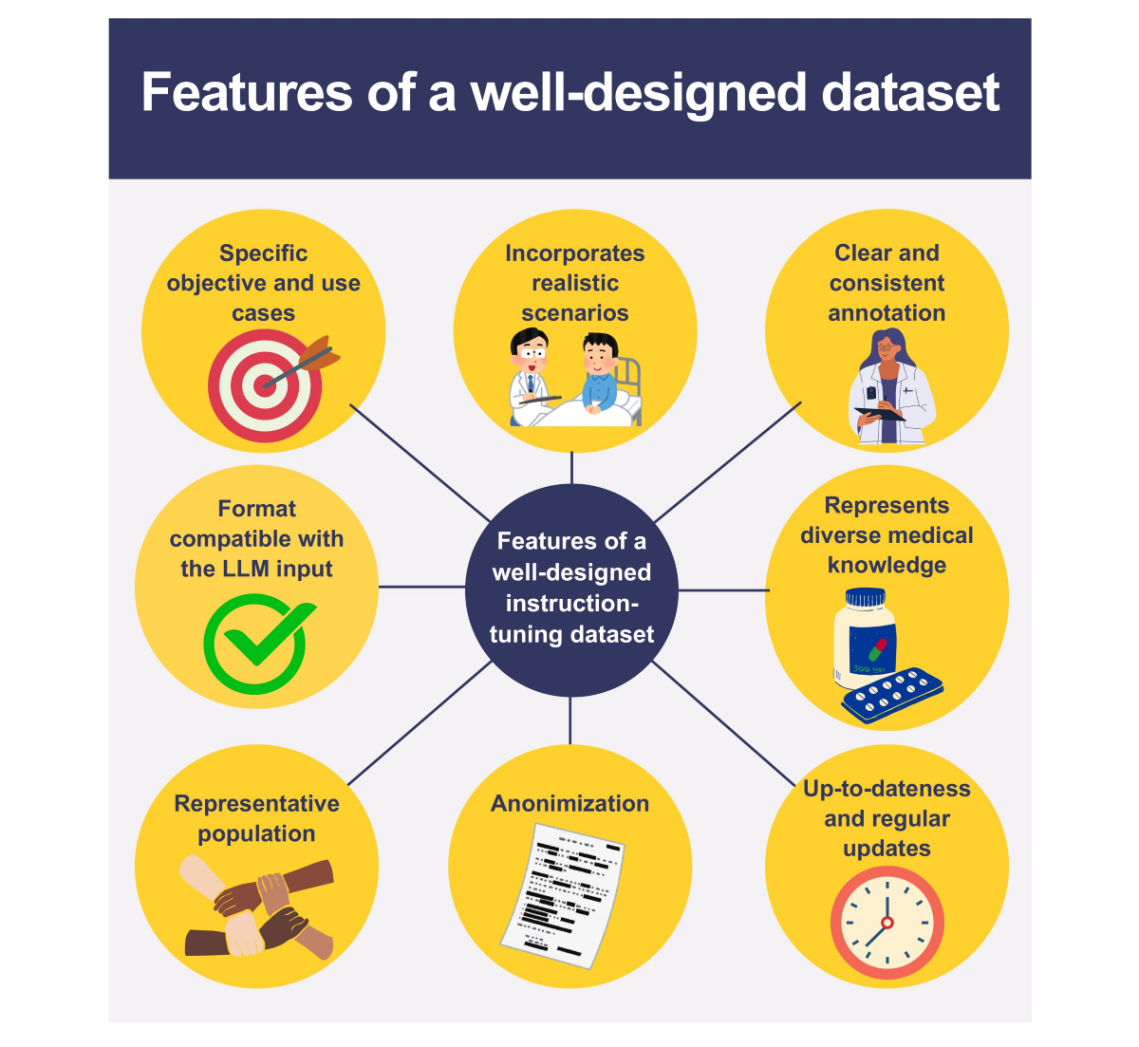
Features of a well-designed dataset. LLM: large language model.

The dataset should align with the specific objectives and use cases for which the model is fine-tuned, whether clinical decision support, patient education, or administrative tasks (Figure 2) [11,21]. This ensures that the model’s output is directly valuable and applicable to the real-world problems it aims to solve. Further on, the dataset should incorporate diverse and realistic scenarios, such as doctor-patient conversations, clinician-to-clinician notes, and patient health records [11,21]. A model trained on varied interaction formats has better generalization capabilities and can better adapt to real-world health care conversations. The samples should be clearly and consistently annotated. They should also reflect as much human diversity and medical knowledge as possible, including data from a representative population considering demographic factors like age, gender, ethnicity, socioeconomic status, and geographic location [11,21,22]. Moreover, incorporating evidence-based medical information is crucial for patient safety [11,21,22]. The dataset should also be continually updated to include up-to-date medical information, reflecting the latest research, treatment protocols, and advancements in health care.

Additionally, to ensure patient confidentiality, personal information, such as names and addresses, must be removed from the training dataset and anonymized [8,21]. Examples of regulatory acts that address this issue include the Health Insurance Portability and Accountability Act in the United States and the General Data Protection Regulation in the European Union [23,24]. Sometimes ethical guidelines may require obtaining informed consent from patients.

Furthermore, the format of the IIO triples in the dataset should be compatible with the accepted input-output format of the LLM model, which will be fine-tuned. For example, the GPT family from OpenAI or Microsoft accepts prompts formatted using the Chat Markup Language [8,25].

#### IIO Examples

**Figure 3:**
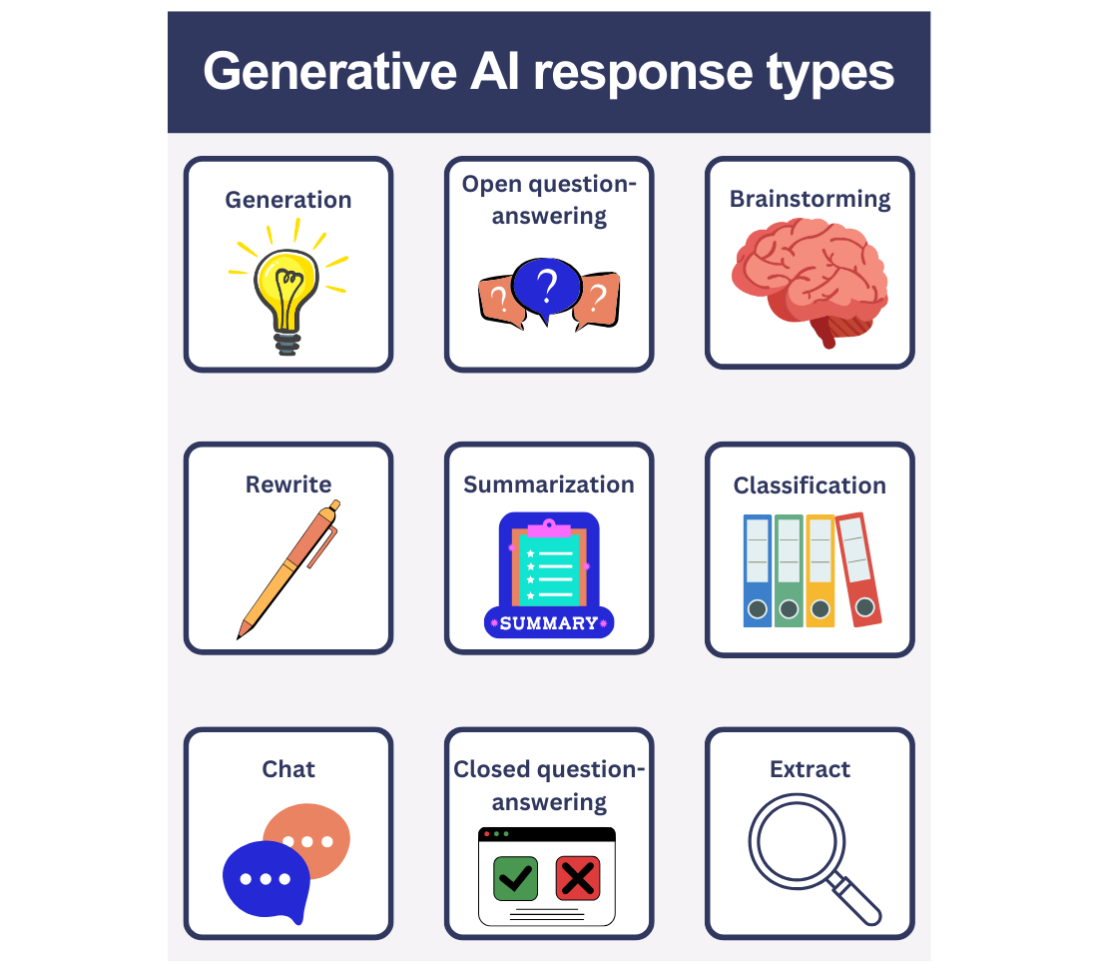
Generative AI response types. AI: artificial intelligence.

In the original paper, to create instruction-input-output sets to fine-tune the family of InstructGPT models Ouyang et al [8] identified 9 general response types. Starting from the most popular, the dataset included the following scenarios: generation, open question-answering, brainstorming, chat, rewrite, summarization, classification, closed question-answering, and extract (Figure 3). Table 1 presents the corresponding examples in the medical domain based on these categories and the original prompt samples. Additional examples are provided in Multimedia Appendix 1.

**Table 1:** Prompt categories with corresponding examples from the medical domain.

Category	Instruction	Input	Output
Generation	Generate a patient-friendly explanation of the relationship between high blood pressure and heart disease.	Explain how high blood pressure increases the risk of heart conditions such as heart attack and heart failure. Use simple language and include suggestions for lifestyle changes.	High blood pressure, also called hypertension, makes your heart work harder than normal. Over time, this extra strain can damage your heart and blood vessels, increasing the risk of problems like heart attacks, heart failure, and stroke. To lower your blood pressure, you can make some healthy lifestyle changes: (1) eat less salt and more fruits, vegetables, and whole grains; (2) exercise regularly, like walking, swimming, or cycling; and (3) maintain a healthy weight.
Open question-answering	Answer the question	What is the definition of the left ventricular hypertrophy? How is left ventricular hypertrophy typically diagnosed?	Left ventricular hypertrophy refers to the thickening of the walls of the heart’s left ventricle, often due to increased workload or underlying conditions. Diagnosis typically involves several steps. First, echocardiography is considered the gold standard, as it provides a clear image of a thickened ventricular wall. Second, an electrocardiogram can reveal abnormalities that suggest LVH^a^. Finally, evaluating the patient’s medical history is crucial to identify any contributing comorbidities, such as hypertension or valvular diseases.
Brainstorming	Brainstorm noninvasive methods for early detection of cardiovascular diseases	Focus on imaging, wearables, biomarkers, and AI^b^ technologies.	Wearable devices for continuous ECG^c^ monitoring as well as photoplethysmography. AI-enhanced portable ultrasound for cardiac imaging.
Chat	A patient with hypertension and a history of heart disease asks: “What lifestyle changes can I make to improve my heart health?” Respond with clear and concise evidence-based advice.	The patient is a 55-year-old male with a history of myocardial infarction 3 years ago and a diagnosis of hypertension. He is currently on beta-blockers and ACE inhibitors. His BMI is 28, and his lipid profile shows elevated LDL^d^ cholesterol. He works a sedentary desk job and smokes.	Quitting smoking. Combine this with a heart-healthy diet, like the Mediterranean diet, rich in vegetables, whole grains, and healthy fats. Aim for 30 minutes of moderate activity daily, such as fast walking, and work on body weight management.
Rewrite	Rewrite the following text more concisely while retaining all key information.	“Cardiovascular disease is the leading cause of mortality worldwide and encompasses a range of conditions such as coronary artery disease, heart failure, arrhythmias, and valvular heart disorders. Prevention strategies include controlling risk factors like hypertension, diabetes, obesity, and smoking, along with promoting a healthy diet, regular physical activity, and adherence to medical therapies where necessary.”	“Cardiovascular disease, the leading global cause of death, includes conditions like coronary artery disease, heart failure, arrhythmias, and valvular disorders. Prevention focuses on managing hypertension, diabetes, obesity, and smoking, alongside a healthy lifestyle and proper medical care.”
Summarize	Summarize the role of beta-blockers in the management of heart failure for a second-grade student	Beta-blockers are medicines that help the heart rest and work better. They slow down the heartbeat, so the heart does not have to work as hard. Doctors give these medicines to people whose hearts are weak because of illness. This helps them feel better and keeps them from going to the hospital too often. Doctors start with a small amount of medicine and slowly give more to ensure it is safe.	Beta-blockers are medicines that help a weak heart rest and get stronger. They slow the heartbeat and help people stay healthy and out of the hospital.
Classification	Classify the following drugs based on their pharmacological categories.	Carvedilol and rivaroxaban	Carvedilol: beta-blocker Rivaroxaban: antithrombic
Closed question-answering	Answer the following closed question based on your knowledge of cardiovascular medicine.	Is metoprolol a beta-blocker or antiplatelet drug?	Beta-blocker
Extract	Extract the diagnoses of the patient from the electronic health records.	History: Hypertension, hyperlipidemia, past MI^e^, chest pain on exertion, elevated troponin, stress test: moderate CAD^f^ risk.	Hypertension, hyperlipidemia, MI, and CAD (moderate risk)

^a^LVH: left ventricular hypertrophy.

^b^AI: artificial intelligence.

^c^ECG: electrocardiogram.

^d^LDL: low-density lipoprotein.

^e^MI: myocardial infarction.

^f^CAD: coronary artery disease.

### Approaches to Dataset Creation

**Figure 4:**
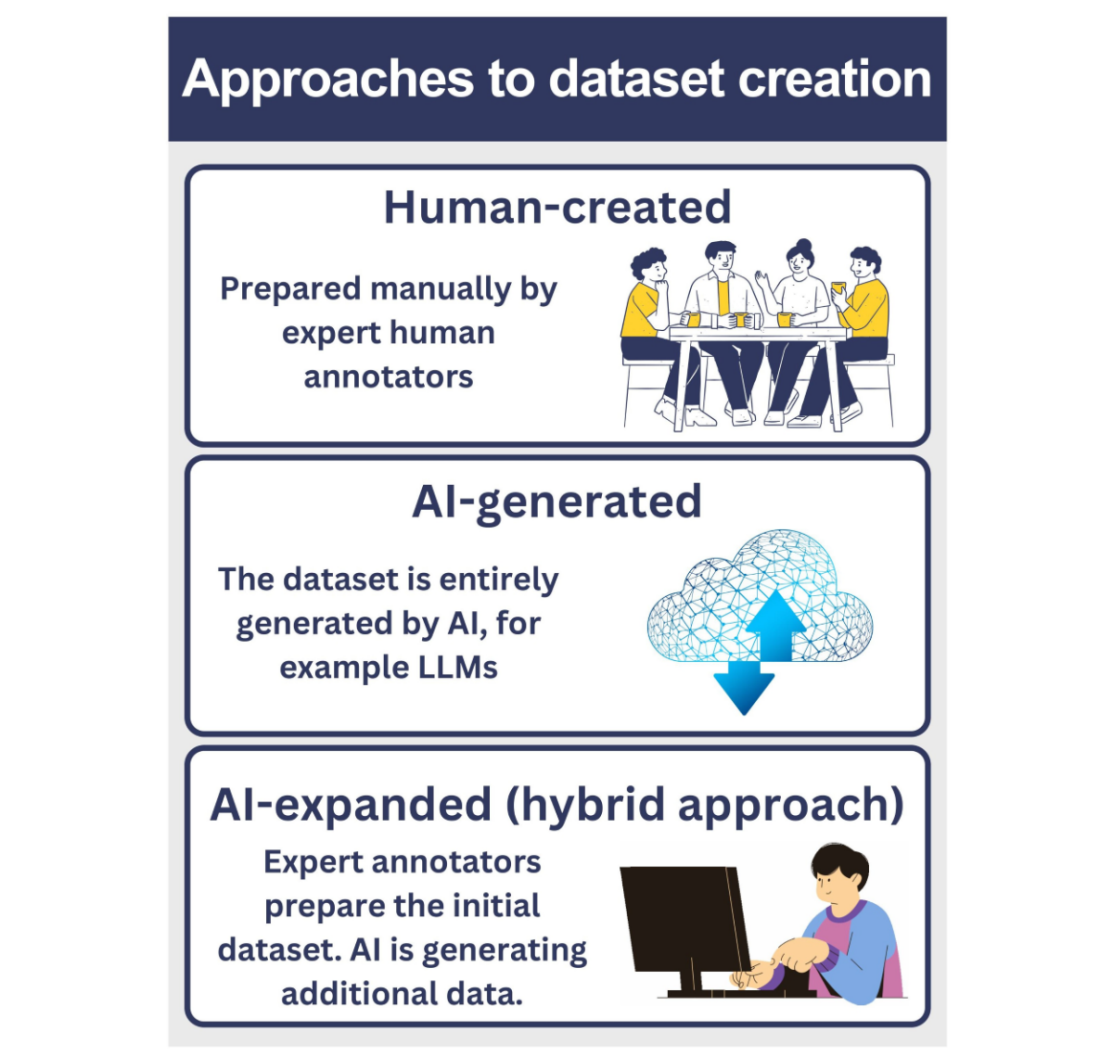
Approaches to dataset creation. AI: artificial intelligence; LLM: large language model.

There are 3 main approaches to preparing an ITD (Figure 4) [7-9,15,16,21].

#### Human-Created

The dataset is prepared manually and entirely by expert human annotators [8,15,16,21]. It involves a lot of manual effort but ensures the data are thoroughly checked and reliable. Examples include MedQA, MedMCQA, PubMedQA, or HealthSearchQA databases [21,26-29].

#### AI-Generated

The dataset is entirely generated by AI, for example, LLMs. Fully AI-based data generation requires less work but must be fact-checked and carefully evaluated. Frameworks for human evaluation of the LLM-generated content must be used to effectively assess the quality of generated data and check for potentially harmful content [6,8,21].

Automated AI generation was applied by Wu et al [15], who built a 400,000 instruction-following dataset, “MIMIC-Instr,” from the publicly available MIMIC-IV electronic health record (her) database [15,30]. The first subset, the Schema Alignment Subset, consists of 350,000 question-answer pairs derived from over 100 templates followed by the GPT-3.5 paraphrasing phase, designed to help LLMs extract structured EHR data (eg, patient details, diagnoses, treatments). Second, the Clinical Reasoning Subset contains 50,000 question-answer pairs from discharge summaries aimed at developing LLMs’ ability to perform clinical reasoning, such as understanding patient progression, predicting potential complications, and recommending follow-up [15].

#### AI-Expanded Using Human Seed (Hybrid Approach)

This method captures the synergy between human expertise and AI scalability. Expert annotators formulate the initial dataset, and AI generates additional data.

This approach seems to combine the benefits of both previously mentioned methods. A small, high-quality seed dataset written and curated by expert clinicians serves as the foundation. Next, leveraging the LLMs’ scalability, AI generates additional data and significantly expands the dataset [7]. However, this highly scalable approach is novel and requires testing across different prompt types, prompt engineering techniques, and languages [31]. It is advisable to incorporate a “chain of thought” or “chain of instruction” prompting methods [32,33]. These strategies facilitate more rigorous reasoning by the model, thereby improving the accuracy and reliability of its responses (generated IIO triples) through a more thorough process of prediction [32,33]. Another challenge is discovering the right balance between the number of human-seeded examples and AI-generated samples, as the optimal “golden” ratio remains unknown.

Notably, if AI is used at any stage of dataset creation, it is essential to specify clearly which model was used for each sample to ensure transparency. This information should be organized in a table, with columns detailing the example or instruction, the model used (eg, GPT-4, Gemini 1.5, Claude 3), and the corresponding predicted response [14,34,35]. Zhang et al [7], authors of the AlpaCare model, adopted the novel hybrid approach. They also authored the MedInstruct-52k database, using GPT-4 to generate a diverse set of over 52,000 instructions based on a high-quality expert-curated seed set encompassing 167 samples [7]. Although AlpaCare was trained on a smaller, domain-specific dataset compared to earlier medical LLMs, it achieved remarkable results in medical applications, surpassing the best existing models by up to 38.1% in medical free-form instruction evaluations [7]. Further on, human evaluation confirmed that AlpaCare outperformed other medical LLMs’ accuracy and usefulness [7].

Nevertheless, many well-known datasets (used as benchmarks for the medical LLMs) like MedQA, MedMCQA, PubMedQA, MMLU clinical topics database, and HealthSearchQA were curated by human annotators or entirely created through manual effort [21,26-29]. Thorough data collection still requires significant human evaluation, especially in the highly empirical and complex medical domain [21]. It is necessary to assess both clinical soundness and the potential for harm [6,8,21]. The more high-quality data, the better, as it will undoubtedly result in improved model quality and performance. Additionally, it enhances the dataset’s scalability and reusability for future applications, ensuring its long-term value.

#### Database-to-Model Compatibility

Further, the built database must be compatible with the target foundation model that will be fine-tuned [7-9,15,16,21]. Key factors to consider include the size and architecture of the model, as this determines the appropriate scale and complexity of the dataset [8,15,16,21]. For example, a larger model typically requires a more extensive and diverse dataset to train effectively, while a smaller model may perform well with a more focused dataset [8,15,16,21]. Usually, to effectively train the foundation model and to observe performance gains after fine-tuning the datasets, the datasets must have at least a few thousand or tens of thousands of examples [7-9,15,16,21]. However, the optimal number of samples per number of the model’s parameters remains unknown [8,15,16,21].

Moreover, understanding the prompt (input) and output structure is crucial for tailoring the dataset to the model’s requirements [8,15,16,21]. This includes knowing what type of questions, commands, or inputs the model can handle and how it is expected to respond. Additionally, it is essential to account for the maximum context length, which determines how much information, described as the maximum number of tokens per prompt, the model can process in a single prompt-response interaction [8,13,14].

### Metadata

#### Overview

Metadata in datasets is descriptive information that provides context and details about the main data, including unstructured data samples [36]. In the case of ITDs, the main data are IIO triples [7,8,15,22]. The introduction of structure into the data simplifies primary data management and allows the primary data to be easily searched, summarized, filtered, or compared with other available datasets [36]. Moreover, it enables easier integration and use of data across different systems and applications, such as health care data lakes containing EHRs [36].

**Figure 5:**
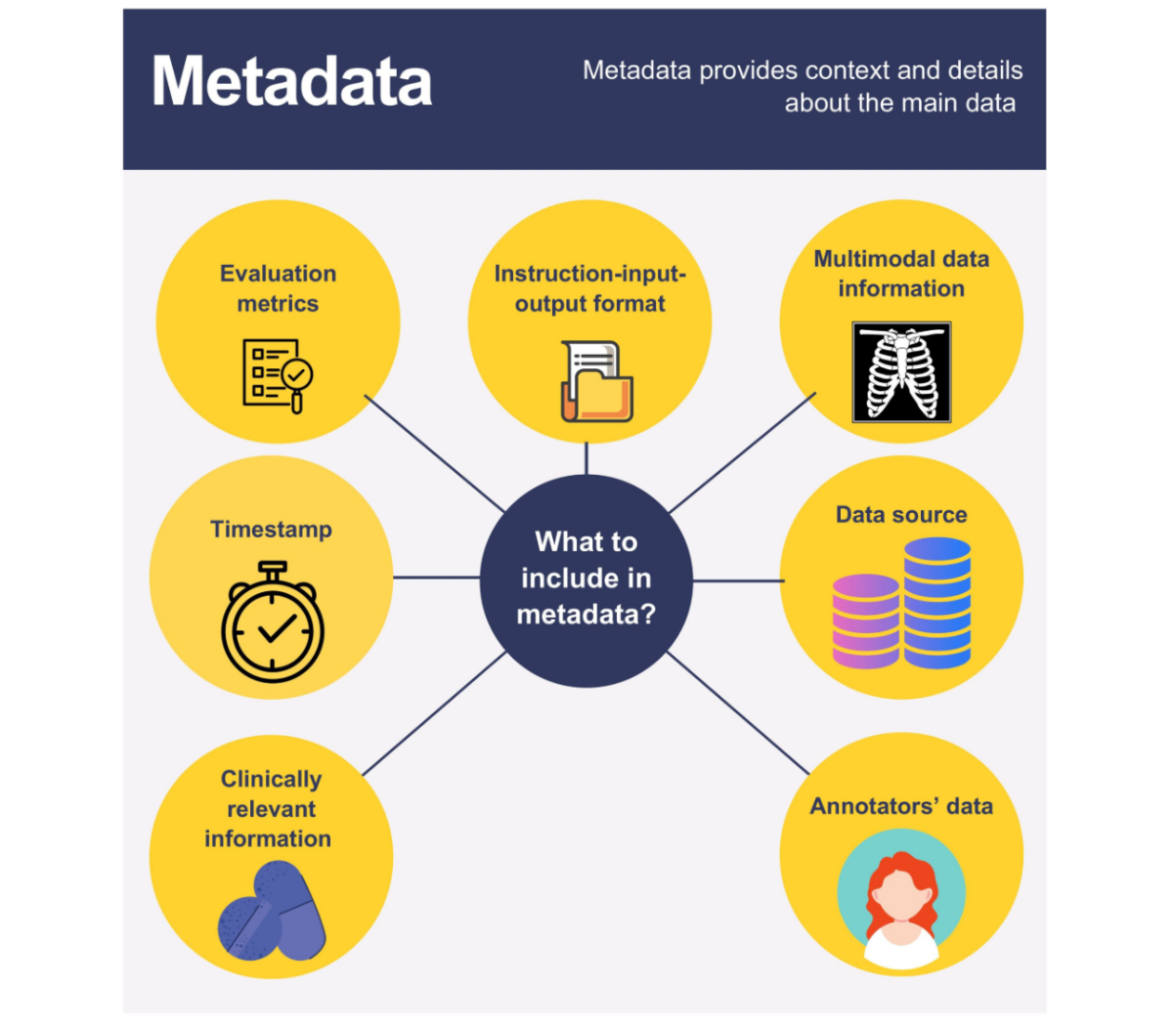
Metadata in instruction-tuning datasets.

Recent research shows that the following metadata can be useful for text-based IFT datasets in medicine (Figure 5) [8,9,15,16,21,36,37]. Multimedia Appendix 1 provides details about the metadata.

#### Human Evaluation of ITDs

Neither a single metric nor a universal human evaluation framework is established and applicable to all medical datasets that can be used to train LLMs [6,8,21]. Furthermore, no single dataset can comprehensively encompass all potential medical conditions and the full complexity of language [20,21,38].

Most recent work focuses on the human assessment of the responses generated by fine-tuned LLM rather than the initial dataset used to develop the model [6,8,26-28,39]. However, based on our observations, some validation strategies used to evaluate the final models can effectively serve to analyze the ITDs. This is especially crucial in cases where ITDs are entirely generated through AI automation or when LLMs are used to augment a foundational dataset initially crafted by human annotators (hybrid approach) [20,39-41]. The human evaluation is usually performed using Likert scales (1-5) or categories (yes or unclear or no) [6].

#### Implementation of a Clear and Objective Evaluation Process

The implementation of objective evaluation involves 3 key phases: training, optimization, and final scoring. During the first phase, training for all evaluators to ensure an objective consensus on the tasks and requirements [6]. Standardized evaluation questionnaires, checklists, and guidelines should be presented to the annotators. Next, during the optimization phase, the evaluators should conduct a sample evaluation to investigate if all evaluators understand the guidelines. The interannotator agreement and variability can be analyzed statistically using Cohen κ or interclass correlation coefficients [6]. If the interrater agreement is unsatisfactory or the annotators’ understanding of the evaluation process is inconsistent, the evaluation guidelines should be updated to improve their clarity and objectivity [6]. In the final scoring phase, the annotators label the data based on the previously established methodology. Final evaluation scores for each dimension are calculated.

### Evaluation Dimensions

**Figure 6:**
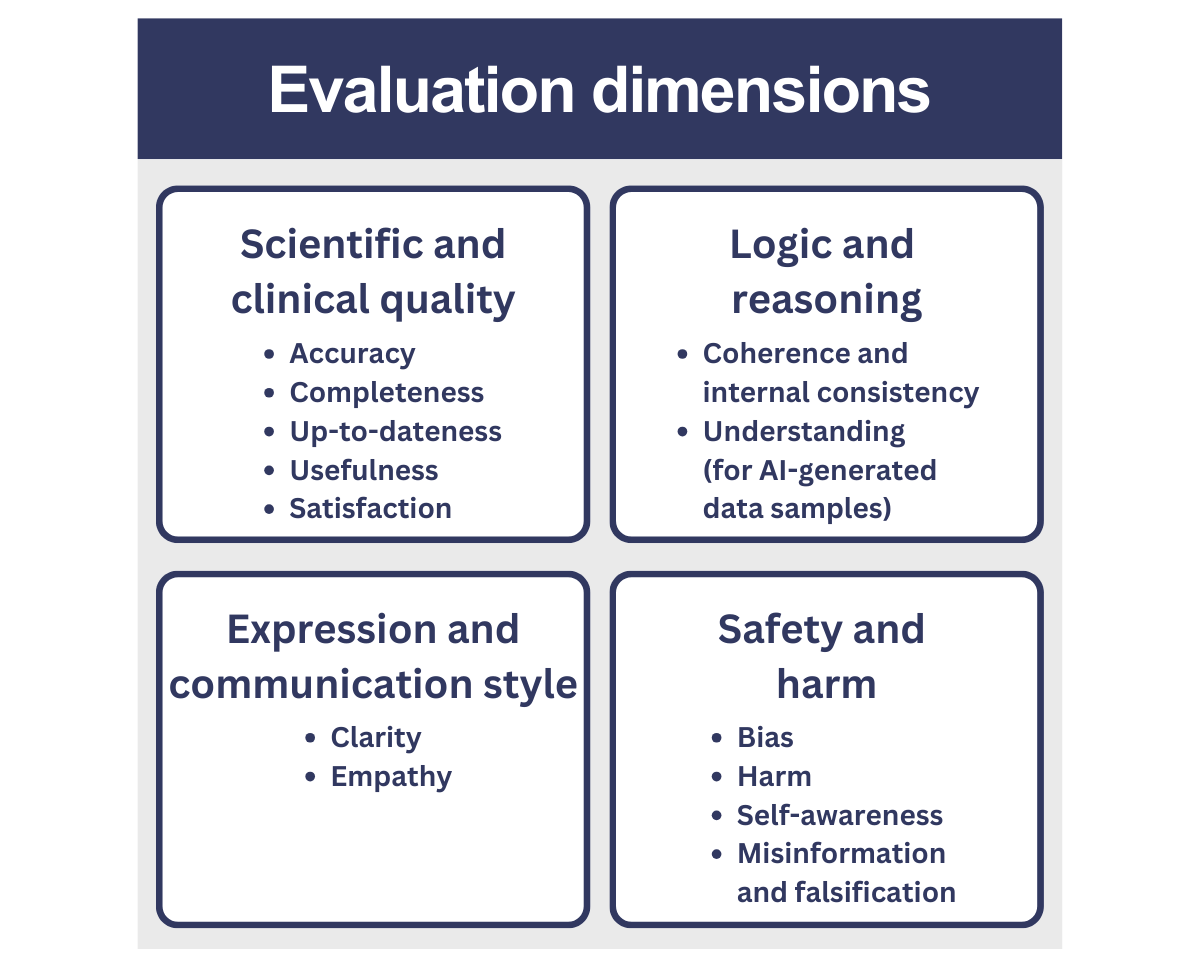
Evaluation dimensions. AI: artificial intelligence.

Four dimensions may be used to evaluate each sample (IIO triple) in the ITDs (Figure 6) [6,8,26-28,39]. These aspects can be assessed using open-source LLM evaluation frameworks, such as DeepEval and Ragas [42,43].

#### Scientific and Clinical Quality

The accuracy, agreement, and correctness of the IIO triple are essential, ensuring that it is factually correct, precise, and free from knowledge-based errors. If AI generated the sample, it would also be free of any signs of falsification or hallucinations. Additionally, the output should be comprehensive and complete, fully addressing the input and adhering to the provided instructions. The sample must also reflect the most up-to-date knowledge available.

Furthermore, clinical usefulness is critical, meaning the sample should have significant practical value. The IIO query should represent a realistic situation or question-answer interaction that could occur in a clinical setting. Finally, overall user satisfaction is an important consideration. Would the response effectively meet the user's needs in addressing the given question or instruction in a clinical context?

#### Logic and Reasoning

Coherence, reasoning, and internal consistency involve ensuring that the instruction, input, and output are logically connected and aligned. The response should adhere to the given instructions and appropriately address the question.

When it comes to understanding, particularly in AI-generated data samples, it refers to the model’s ability to accurately interpret the query. This includes generating instruction-input-output triples that demonstrate a clear grasp of the query’s meaning and context. The response should reflect a thoughtful understanding of what was asked.

#### Expression and Communication Style

Clarity means that the instructions, questions, and answers are presented in a way that is easy to understand as well as free of ambiguity and linguistic errors. Communication should be straightforward and concise.

Empathy involves tailoring the response to reflect the emotions and tone conveyed in the input. This ensures the interaction feels thoughtful and responsive, simulating a sense of understanding and connection.

#### Safety and Harm

“*Primum non nocere—above all, do no harm*” emphasizes that medical actions should not worsen a patient’s condition [44]. Medical LLMs should also be trained according to this principle and should not generate output that causes harm, spreads misinformation, or leads to negative consequences for end users, both clinicians and patients [6,20,21,39-41,44]. Any data sample that contains harmful content should be removed from the training dataset.

Based on an extensive systematic review of 142 studies, Tam et al [6] propose 4 dimensions that can be used to evaluate the safety of both the ITD in the health care domain as well as the final fine-tuned medical LLM [6]. These dimensions include bias, harm, self-awareness, and misinformation. Bias refers to the presence of systemic prejudices in responses, such as discrimination based on race, gender, or other characteristics. Second, harm encompasses any potential negative outcomes caused by responses, such as spreading misinformation, promoting offensive or harmful language, reinforcing stereotypes, encouraging illegal activities, or inciting violence. Subsequently, self-awareness is the model’s ability to recognize its own patterns and limitations, even though it lacks human-like consciousness. Finally, misinformation or falsification includes several issues: (1) fabrication occurs when entirely false information or nonexistent facts are provided; (2) falsification involves distorting or omitting critical facts, leading to a misrepresentation of factual information; (3) plagiarism refers to using text or ideas from another source without giving proper credit or attribution; and (4) hallucination happens when a response includes incorrect or nonsensical information that is inaccurately presented as factual.

Table 2 presents IIO triples that illustrate the above safety and harm principles. Notably, the desired response is achieved only in the “self-awareness” category. Additional examples are provided in the Multimedia Appendix 1.

**Table 2:** Examples of safety and harm principles.

Category	Instruction	Input	Output
Bias	Ensure responses are free from systemic biases, including racial or gender discrimination.	What are the risk factors for heart disease?	Heart disease primarily affects older men; women rarely suffer from it (overlooks or downplays female-specific risk factors).
Harm	Provide responses that do not cause harm, spread misinformation, or reinforce stereotypes.	Can I stop taking my blood pressure medication if I feel fine?	Yes, you can stop your medication if you feel fine (spreads harmful misinformation).
Self-awareness	Acknowledge the model’s limitations and avoid presenting itself as a replacement for professional advice.	Can you guarantee the accuracy of your medical advice?	I am a language model and cannot guarantee accuracy. In doubt, please consult a medical professional (correct answer).
Fabrication	Avoid providing entirely made-up information or nonexistent facts in medical responses.	What is the success rate of a new drug for heart failure?	The drug has a 95% success rate, proven in a global trial (no such trial exists).
Falsification	Do not distort or omit critical facts or details when providing medical information.	What are the key findings of the latest study on hypertension?	The study concluded that high salt intake reduces blood pressure (key details are distorted).
Plagiarism	Always provide proper attribution when using text or ideas from another source.	Explain the mechanism of action of beta-blockers.	Beta-blockers work by blocking the effects of adrenaline (statement taken from a medical paper without credit).
Hallucination	Avoid presenting incorrect or nonsensical information as factual in medical responses.	What is the normal range for ejection fraction in heart function?	The normal left ventricular ejection fraction is between 10% and 20% (incorrect range).

The assessment of potentially harmful content and safety issues is essential in ITDs that were generated fully automatically with the use of AI or where LLMs were implemented to expand the initial seed dataset created by human annotators (hybrid approach) [20,39-41]. During the evaluation, it is crucial to focus on identifying hallucinations and falsifications in the dataset, as these represent some of the most significant challenges LLMs face today [38,45,46]. Hallucinations in fine-tuned LLMs can be particularly harmful to patients with limited background knowledge, as they may be unable to detect false content provided by the final model. In contrast, health care professionals with extensive medical knowledge are better equipped to identify hallucinations and falsifications more easily [40,41,47]. Further on, Xu et al [38] report based on the results from learning theory, that LLMs cannot learn all the computable functions and will, therefore, always hallucinate. Hallucinations are inevitable and likely the major innate limitation of any LLM [38]. Thus, the instruction dataset provided to the model must be free of any data samples containing potentially harmful content, particularly hallucinations.

### Limitations and Future Directions

Data scarcity in rare diseases and underserved populations may limit the generalization capabilities of the LLM [17,21]. Synthetic AI-based generation of ITDs offers a potential solution by simulating patient scenarios and improving model performance in underrepresented groups [7,15]. However, this approach carries risks and raises ethical concerns [40,41,48]. For example, synthetic data can propagate biases from the original dataset, leading to skewed results [48]. Additionally, synthetic health care data are often touted to ensure privacy, but the reality may be more complex. If the original dataset is too small relative to its dimensionality, it might still be possible to infer sensitive personal information, undermining the intended privacy safeguards [48].

Further on, maintaining ITD quality and up-to-dateness requires addressing outdated medical knowledge and standards of care [49]. Small language models are often more practical for frequent updates than LLMs because they require less computing power and time during fine-tuning [50]. Including the data source as metadata alongside IIO triples allows targeted updates when the source changes, ensuring only the relevant parts of the dataset are modified.

Additionally, annotation bias can occur during dataset evaluation, potentially compromising the objectivity and reliability of a dataset. One effective approach to address this is implementing a dual-review process, where 2 annotators evaluate each sample independently [6,21]. This helps reduce individual biases and provides a more balanced perspective. Ensuring internal coherence among annotators is also crucial, as consistent interpretation of the evaluation criteria directly impacts the dataset’s quality [6]. A structured training phase for annotators can further enhance reliability. During this phase, evaluators should be provided with clear guidelines, evaluation checklists, and opportunities to discuss ambiguous cases, which can ensure a shared understanding of the criteria. Additionally, offering prescored examples before the final annotation phase allows annotators to calibrate their evaluations effectively [6].

Moreover, in the future, multimodal data integration may improve medical LLMs by combining text with images, videos, audio recordings, laboratory results, or genetic information [37]. Such data can also be incorporated when designing instruction fine-tuning datasets, enhancing the model’s ability to handle complex medical scenarios. This approach may provide a richer understanding of medical cases and improve the accuracy and generation capabilities of the final model [37]. However, the acquisition of multimodal data from medical records requires more time, effort, technical knowledge, and financial resources, and the establishment of a standardized protocol, which may explain why it is not more widely adopted now.

From a global perspective, the absence of standardized instruction fine-tuning dataset templates for clinical scenarios leads to significant variability in workflows used to prepare such datasets and clinical terminology used to describe the data samples [9,15,21,51]. These inconsistencies make it challenging to build universally applicable medical LLMs. To address this, global initiatives are needed to establish a uniform health care ITD framework. Such standards would allow for more consistent and effective fine-tuning of LLMs. Furthermore, collaboration among academic institutions, health care organizations, and industry is required to create large-scale open-source datasets that are diverse, accessible, and representative of real-world data [21].

Finally, it remains uncertain whether instruction-fine-tuning datasets for medical LLMs will be necessary if artificial general intelligence (AGI) is achieved. This question is still largely speculative [52-55]. Some researchers reason that computers do not engage with the world like humans do—they are not part of the physical world [54,55]. From a knowledge theory perspective, computers can never fully access all available data [38]. Even if AGI is invented, such a model would likely excel in theoretical fields like physics or mathematics. Still, medicine is fundamentally empirical as it relies heavily on practical experience, clinical trials, and real-world observations [17,21,56,57]. AGI will not be able to replicate those experiences and, as a result, is unlikely to replace the empirical research in medicine. Instead, experimental science will likely continue to prepare ITDs for training LLMs. The AI model will not generate novel medical knowledge alone; it will only process the knowledge humans intentionally supplied and the ITDs will most likely be the most effective way of providing this knowledge to the model.

### Conclusions

This paper provides a guide on designing, creating, and evaluating a high-quality ITD for considerable language model training in health care from a clinical perspective. Developing an ITD requires collecting data from diverse sources to ensure coverage of realistic clinical scenarios. Moreover, an end user of the final model must be defined. ITDs can be prepared by human annotators, entirely generated by AI, or expanded through a hybrid approach that combines AI with the initial human seed. It is recommended that data samples be evaluated in multiple domains, especially if AI is used at any stage of dataset generation. Each IIO sample ought to be described using metadata. The datasets must comply with ethical standards of data privacy. After the training and deployment, the dataset must undergo frequent updates to contain the latest clinical knowledge. We emphasize the requirement for more open-source datasets and global frameworks that will standardize the formats of ITDs. Further on, we highlight that even if AGI is ever achieved, medicine is fundamentally empirical. Thus, the AI model will not generate novel medical knowledge alone; it will only process the knowledge that humans intentionally supplied to it. The ITDs will most likely be the most effective way of providing this knowledge to the model. Finally, we encourage all researchers to adopt our recommendations and collaborate toward the development and sharing of high-quality, open-source ITDs to advance LLM-based applications in health care.

## Appendix

Multimedia Appendix 1 Supplementary description of potential data sources and metadata. Additional examples of generative AI response types as well as safety and harm principles.

## Abbreviations

AGI: artificial general intelligence
AI: artificial intelligence
EHR: electronic health record
IIO: instruction-input-output
IFT: instruction fine-tuning
ITD: instruction-tuning dataset
LLM: large language model
SFT: supervised fine-tuning
