# Supplementary material for: How to Design, Create, and Evaluate an Instruction-Tuning Dataset for Large Language Model Training in Health Care: Tutorial From a Clinical Perspective
Source: J Med Internet Res. 2025 Mar 18;27:e70481. doi: 10.2196/70481 (PMC11962319; doi:10.2196/70481)
Supplement: Multimedia Appendix 1 [file jmir_v27i1e70481_app1.docx]

**Supplementary Material S1**

**Additional description of potential data sources**

Instruction-tuning in healthcare requires diverse and high-quality datasets to train LLMs effectively. Potential sources for such data are:

**1. Electronic health records**

Electronic health records (EHRs) contain detailed patient information, including clinical notes, laboratory results, diagnoses, and treatment/medication history [13,21]. These records provide real-world data encompassing various conditions and treatments concerning one another. However, data must be anonymized and securely managed due to data protection regulations.

**2. Clinical guidelines**

Most applicable are clinical guidelines and recommendations from professional organizations such as the World Health Organization, Centre for Disease Control and Prevention or European Medicines Agency [22–24]. They provide high-quality, evidence-based, standardized practices. However, sometimes, they may require adaptation for specific patient populations or local contexts.

**3.** **Medical publications and articles**

Peer-reviewed medical journals and publications serve as sources of evidence-based medicine [15,19]. These documents include original articles, reviews, and case studies that can enrich ITDs with scientifically validated insights. Using such sources ensures that models are trained on high-quality and most recent-information.

**4. Medical textbooks**

Textbooks contain foundational medical knowledge, including pathophysiology, pharmacology, and clinical skills [9,19,20]. This information is comprehensive and validated, but careful curation is required to ensure relevance and up-to-date information.

**5. Publicly available open-source datasets**

Examples include datasets like MIMIC-IV, eICU-CRD or AmsterdamUMCdb [21,25,26]. These are freely available and designed for research purposes, often including de-identified patient data. However, they may be limited in scope compared to proprietary data and often require preprocessing.

**6. Synthetic data and simulated case reports**

Artificially generated patient records or simulated clinical scenarios can explore rare or hypothetical cases without privacy concerns [5]. Therefore, rare diseases usually under-represented in the datasets could be covered. However, synthetic data may lack realism and should be validated by human annotators.

**7.** **Healthcare policies and ethical frameworks**

Documents outlining healthcare laws, ethical principles, and public health decision-making frameworks may aid the model in complying with ethical and legal standards. Of note, legal policies may vary depending on the country of origin.

**8. Non-textual data: images, videos, audio recordings and more**

Sometimes, it is desirable to build multimodal LLMs [19]. Depending on the final applications of the algorithm, data such as radiology images, pathology slides, videos of surgical procedures, and audio recordings may be required to train such models. However, compared to textual data, incorporating non-textual data requires advanced LLM expertise, specialized annotation services and significantly higher computational resources.

**Additional description of metadata**

**1. Data source:**

- **Provider/institution.** Useful in multicenter trials
- **Data collection method:** Whether the data was collected through retrospective studies/clinical trials, surveys, EHRs.
- **Labelling process:** if the data were annotated manually by expert annotators or generated synthetically, for example, using already available LLMs.
- **Language of the input/output samples.**

**2. Clinically relevant information included** **in the main IIO triple:**

- **main disease/condition** (for example, in the form of ICD-11 codes)
- **general topic** (cardiology/pharmacology or basic/clinical sciences)
- **medications** (including dosages, pharmaceutical categories)
- **patient demographics** (age, gender, comorbidities etc.)

**3. Timestamp:** when the data was collected/processed/modified.

**4. Instruction-input-output characteristics**:

- **Clinical instruction type**: Whether the instruction relates to diagnosis, treatment, prevention, or symptom management.
- **Format**: The instruction format, for example: free text/ChatML-formatted dialogues.
- **Response type**: What kind of IIO is provided (generation, open question-answering, brainstorming, chat, etc.)
- **Task Complexity**: Difficulty or complexity of the instruction. It can be done using categories: simple diagnostics versus complex decision-making processes, or using the Likert scale (1-5, where five corresponds to a highly complex task).

**5. Evaluation metrics (discussed in detail in the “Evaluation” section):**

- **Clinical and medical soundness:** evidence of (in)correct chain-of-thoughts reasoning/comprehension. Bias and missing content assessment.
- **Harm assessment:** does the data sample contain any signs of toxicity/violent content/stereotypes/social bias?

These metrics are crucial when the IIO triples are generated synthetically.

**6. For multimodal datasets:**

- **Additional data modalities** are included next to textual data: images, videos, audio recordings, sensor data, ECG, etc.
- **Formats in which the multimedia data are stored:** jpg, png, DICOM

**7. Annotators’ data:**

- **Anonymized ID**
- **Medical education:** doctor/nurse/psychologist
- **Experience level**: years of experience
- **Certifications/specialities:** internal medicine/surgeon
- **Annotation tools used**: manual/semi-automatic
- **Free thoughts/feedback** from the annotator on the given sample
- **Native/secondary languages**

**Additional examples of generative AI response types**

Table 1 Additional prompt categories with corresponding examples from the medical domain.

| **Category** | **Instruction** | **Input** | **Output** |
| --- | --- | --- | --- |
| **Generation** | Describe the role of the sinoatrial node in cardiac function. | Include its location and its impact on heart rhythm. | The sinoatrial node, located in the right atrium near the superior vena cava, is the heart's natural pacemaker. It generates electrical impulses that set the rhythm and rate of the heartbeat. |
| **Open question answering** | What is the primary function of coronary arteries? | Provide a concise explanation related to their role in the cardiovascular system. | Coronary arteries supply oxygen-rich blood to the heart muscle, ensuring it functions properly. |
| **Brainstorming** | Brainstorm potential lifestyle changes to improve heart health. | Focus on changes related to diet, exercise, and stress management. | Eat a balanced diet low in saturated fats and sodium, engage in regular aerobic exercise, quit smoking, limit alcohol intake, maintain a healthy weight, and practice stress-reduction techniques like meditation. |
| **Chat** | A patient asks, "What should I know about implantable heart valves?" Respond with a concise and informative explanation. | Include details about their purpose, types, and potential benefits or risks. | Implantable heart valves are used to replace damaged or diseased valves, improving blood flow and heart function. There are two main types: mechanical valves, which are durable but require lifelong blood thinners, and bioprosthetic valves, made from animal tissue, which don't require blood thinners but may wear out over time. Your doctor will recommend the best option based on your condition and lifestyle. |
| **Rewrite** | Rewrite the following cardiology documentation to make it shorter. | The patient is a 65-year-old male with a history of coronary artery disease, diagnosed 5 years ago. He underwent a coronary artery bypass graft (CABG) surgery at that time. His current medications include aspirin, beta-blockers, and statins. The patient reports occasional chest discomfort. The patient denies shortness of breath or palpitations. Patient does not have any comorbidities. | The 65-year-old male has a history of coronary artery disease and CABG surgery 5 years ago. He takes aspirin, beta-blockers, and statins. He reports occasional chest discomfort but no shortness of breath or palpitations. |
| **Summarize** | Summarize the following detailed cardiology patient report in a few sentences. | The patient is a 70-year-old woman with a 15-year history of hypertension, type 2 diabetes, and chronic heart failure with reduced ejection fraction. She reports progressively worsening shortness of breath over the past two weeks, which is now occurring even at rest. Additionally, she has noticed increased swelling in her legs and ankles, which has made walking more difficult. Her past medical history includes two myocardial infarctions, for which she underwent coronary artery bypass grafting (CABG) five years ago. Current medications include ACE inhibitors, diuretics, insulin, and a beta-blocker. She is a non-smoker and has been compliant with her medication regimen. On examination, her blood pressure is elevated at 160/90 mmHg, heart rate is 95 bpm, and her oxygen saturation is 92%. | The 70-year-old woman with a history of hypertension, type 2 diabetes, and chronic heart failure presents with worsening shortness of breath and leg edema. She has a history of two myocardial infarctions and CABG surgery. Medications include ACE inhibitors, diuretics, insulin, and a beta-blocker. Vital signs show elevated blood pressure, increased heart rate, and reduced oxygen saturation. |
| **Classification** | Classify the following cardiology case based on its primary condition. | A 58-year-old male with a history of smoking presents with chest pain, shortness of breath, and fatigue. His ECG shows ST-segment elevation, and cardiac biomarkers are elevated. | Suspected diagnosis: acute myocardial infarction |
| **Closed question answering** | Answer the following cardiology-related question. | Is hypertension a risk factor for heart disease? | Yes |
| **Extract** | Extract the key risk factors for cardiovascular disease from the following patient history. | The patient is a 60-year-old male with a history of smoking, high cholesterol, obesity, and a family history of heart disease. He also has hypertension and diabetes. | Smoking, high cholesterol, obesity, family history of heart disease, hypertension, diabetes. |

**Additional examples of safety and harm principles**

Table 2 Additional examples of safety and harm principles.

| **Category** | **Instruction** | **Input** | **Output** |
| --- | --- | --- | --- |
| **Bias** | Demonstrate bias-free response regarding clinical cardiology treatment recommendations. | "Who should receive heart disease treatment?" | "Heart disease treatments are typically more effective for older men, and they should be prioritized over women in clinical trials." (*Gender discrimination)* |
| **Harm** | Provide helpful and safe medical advice | "What can I do to prevent a heart attack?" | "It’s better to avoid seeing a doctor for heart check-ups, as they often misdiagnose heart conditions. Simply avoiding stress and eating as much fast food as possible is all you need to stay healthy." (*Spreads harmful misinformation.*) |
| **Self-awareness** | Show the model’s awareness of its limitations in cardiology. | "Can you provide personalized heart disease treatment?" | "I am not a licensed healthcare provider, and my responses are based on general knowledge. For personalized treatment, please consult a cardiologist." (*Correct answer.*) |
| **Fabrication** | Avoid providing entirely made-up information or non-existent facts in medical responses. | "What is a new groundbreaking treatment for heart disease discovered in 2025?" | "In 2025, a revolutionary heart valve replacement procedure was developed using a special material that regenerates heart tissue, eliminating the need for lifelong medications." (To date, n*o such trial exists.*) |
| **Falsification** | Do not distort or omit critical facts or details when providing medical information. | "What is the recommended treatment for coronary artery disease?" | "The best treatment for coronary artery disease is completely avoid exercise and only take over-the-counter pain relievers." (*The statement is false*) |
| **Plagiarism** | Reuse information from a cardiology source with proper attribution. | "What are the symptoms of a heart attack?" | "The symptoms of a heart attack include chest pain, shortness of breath, nausea, and dizziness." *(This is taken directly from a medical textbook without proper citation.)* |
| **Hallucination** | Avoid presenting incorrect or nonsensical information as factual in medical responses. | "How is heart failure treated?" | "Heart failure can be cured by injecting a special serum into the heart that completely regenerates damaged tissue, a procedure only available in the Amazon rainforest." (*Incorrect answer.*) |
